# Percutaneous full-endoscopic bullet removal from the C2 vertebra in a pediatric patient

**DOI:** 10.3171/2024.1.FOCVID23230

**Published:** 2024-04-01

**Authors:** Özkan Çeliker, Yücel Doğruel

**Affiliations:** 1 Department of Neurosurgery, Denizli Sağlık Hospital, Denizli, Turkey; and; 2 Department of Neurosurgery, Tunceli State Hospital, Denizli, Turkey

**Keywords:** bullet, endoscopic, gunshot, spine

## Abstract

Gunshot injuries (GSIs) to the spine constitute approximately 17% to 21% of all traumatic spinal injuries, with the cervical spine being the second most frequently affected region. There is a lack of approved algorithms for patients with GSIs to the spine. Surgical intervention is controversial; however, it is generally considered in cases involving neurological deterioration with incomplete deficit, externalized liquor fistula, instability, installed toxicity, and risk of migration. Detailed information on pediatric patients is limited, primarily due to the predominance of adult patients. This study presents the full-endoscopic removal of a bullet in the C2 vertebra of a pediatric patient.

The video can be found here: https://stream.cadmore.media/r10.3171/2024.1.FOCVID23230

## Transcript

This video demonstrates percutaneous full endoscopic bullet removal from the C2 vertebra in a pediatric patient.

### 0:30 Clinical Presentation and Neurological Examination.

A 12-year-old female patient was admitted to the emergency department with a gunshot wound. The patient was conscious, hemodynamically stable, and showed no deficits on the neurological examination. The gunshot wound was located above the nose, just below the nasion.

Cervical computed tomography of the patient revealed a bullet that entered the C2 vertebral corpus from the anterior aspect and lodged in the right C2 pedicle.

### 1:01 Rationale for Surgical Intervention.

Surgical intervention was chosen due to the potential risks associated with the mobilization of the cervical bullet, as well as concerns regarding long-term lead intoxication and possible developmental effects in the growing patient.^1,2^

### 1:20 Surgical Plan.

Our surgical plan is to perform a posterior cervical interlaminar paramedian approach between the C2 and C3 vertebrae from the right side in the prone position.^3^ The interlaminar approach will be performed by inserting the endoscope, followed by opening a small window through partial drilling of the C2 lamina inferiorly and the C3 lamina superiorly from the right side.

Care must be taken to avoid injury to the vertebral artery on the lateral side and the dura and spinal cord on the medial side, as they are at risk during the procedure.

### 2:00 Surgical Procedure.

After the induction of general anesthesia, the patient was positioned prone. The incision and endoscope entry point were determined with the guidance of a needle under fluoroscopic control in anterior-posterior and lateral views.^4^

These fluoroscopic images demonstrate the use of the needle to determine the surgical trajectory. After determining the surgical projection, the endoscope was inserted, and a small bony window was opened with partial drilling of the right-sided C2 lamina inferiorly and C3 lamina superiorly. Following this, the right pedicle of C2 was minimally drilled to expose the bullet completely. Subsequently, the bone around the bullet was carefully removed using a high-speed drill. This circumferential maneuver was performed to create sufficient space, enabling the subsequent use of forceps for bullet retrieval. Following this, the access point was expanded using a Kerrison rongeur.

### 3:34 Bullet Removal.

After opening adequate space, the bullet was carefully retrieved using forceps. The surgical cavity was subsequently irrigated with saline solution. Hemostasis was achieved, and the operation was completed.

### 4:06 Postoperative Period.

Postoperative cervical CT revealed no complications.

The surgical duration was 50 minutes, and no significant bleeding occurred during the operation. The postoperative period was uneventful, and the patient was discharged with a semirigid cervical collar, exhibiting no neurological deficits or complications.

The patient did not undergo emergency axial spine stabilization because the dens was intact from the left side, and the left C2 pedicle was intact. The decision to apply axial spine stabilization to the patient will be based on the development of instability findings during the follow-up.^5^

## Disclosures

The authors report no conflict of interest concerning the materials or methods used in this study or the findings specified in this publication.

## Author Contributions

Primary surgeon: Çeliker. Editing and drafting the video and abstract: Doğruel. Critically revising the work: Doğruel. Reviewed submitted version of the work: Doğruel. Approved the final version of the work on behalf of both authors: Doğruel. Supervision: Doğruel.

## Supplemental Information

### Patient Informed Consent

The necessary patient informed consent was obtained in this study.
